# Soyeom-Jetong mixture attenuates NLRP3 inflammasome-mediated inflammation

**DOI:** 10.3389/fphar.2026.1836840

**Published:** 2026-06-16

**Authors:** Qianying Ye, Sojung Lee, Yeon-Cheol Park, Myung-Seo Kim, Wonnam Kim, Jong-Sik Jin, Dong-Keun Kim, Seoyeon Jang, Sumin Lee, Nahyun Lee, Seongjong Lee, Byung-Kwan Seo, Man S. Kim, Yeonhak Kim, Duckgun An, Sangsu Seo, Donggyu Lee, Hyungyu Kim, Yong Hwan Park, Yoonsung Lee, Yong-Hyeon Baek

**Affiliations:** 1 Department of Biomedical Science and Technology, Kyung Hee University, Seoul, Republic of Korea; 2 Department of Microbiology, Ajou University School of Medicine, Suwon, Republic of Korea; 3 Department of Biomedical Sciences, Graduate School of Ajou University, Suwon, Republic of Korea; 4 BK21 R&E Initiative for Advanced Precision Medicine, Ajou University School of Medicine, Suwon, Republic of Korea; 5 Department of Acupuncture and Moxibustion, Joint and Spine Center, Kyung Hee University College of Korean Medicine, Kyung Hee University Hospital at Gangdong, Seoul, Republic of Korea; 6 Department of Orthopaedic Surgery, Shoulder and Elbow Clinic, Kyung Hee University School of Medicine, Kyung Hee University Hospital at Gangdong, Seoul, Republic of Korea; 7 Division of Pharmacology, School of Korean Medicine, Pusan National University, Yangsan, Republic of Korea; 8 Department of Oriental Medicine Resources and LED Agri-bio Fusion Technology Research Center, Jeonbuk National University, Iksan, Republic of Korea; 9 College of Pharmacy, Jeonbuk National University, Jeonju, Republic of Korea; 10 Clinical Research Institute, Kyung Hee University Hospital at Gangdong, School of Medicine, Kyung Hee University, Seoul, Republic of Korea; 11 East-West Bone and Joint Disease Research Institute, Kyung Hee University, Seoul, Republic of Korea; 12 Department of Clinical Practice, Jahwang Korean Medicine Hospital, Gyeonggi-do, Republic of Korea; 13 Department of Research and Development, KSCP, Gyeonggi-do, Republic of Korea; 14 Department of Academic Research, Korea Society of Clinical Pharmacopuncture, Seoul, Republic of Korea; 15 Center for Space Biomedical Sciences, NEXUS Institute, Kyung Hee University, Seoul, Republic of Korea

**Keywords:** anti-inflammatory, inflammasome, NLRP3, Soyeom-Jetong, zebrafish

## Abstract

Background. Soyeom-Jetong (SJ) is a traditional East Asian polyherbal formulation composed of *Paeonia lactiflora* Pallas, *Glycyrrhiza inflata* Batal, and *Corydalis yanhusuo* W.T. Wang, traditionally used for treating inflammatory disorders. Given the central role of the NLRP3 inflammasome in inflammation-related pathologies, SJ is of particular interest as a potential anti-inflammatory modulator. However, the molecular mechanisms underlying the anti-inflammatory effects remain unclear. This study aimed to evaluate the anti-inflammatory efficacy of SJ in a lipopolysaccharide (LPS)-induced zebrafish model and determine its regulatory effects on the NLRP3 inflammasome *in vitro*. Methods. Inflammation was induced in zebrafish embryos via LPS exposure and tail amputation. The anti-inflammatory effects of SJ were assessed through Sudan Black B staining and whole-mount *in situ* hybridization to detect neutrophil infiltration. *In vitro* assays were performed to investigate the regulatory effects of SJ on NLRP3 inflammasome activation. Results. SJ significantly reduced neutrophil accumulation in the caudal hematopoietic tissue of LPS-exposed zebrafish and the injury site of zebrafish embryos with amputated tails. It also modulated inflammatory gene expression, including NLRP3 and proinflammatory cytokines. *In vitro,* SJ suppressed NLRP3 inflammasome activation. Conclusion. SJ exhibits anti-inflammatory activity by modulating neutrophil inflammation and suppressing NLRP3 inflammasome activation *in vitro*. These findings provide experimental support for the pharmacological activity of SJ and suggest its potential as an anti-inflammatory candidate for further investigation.

## Introduction

Inflammation is a fundamental defense mechanism that protects the host from infection and tissue damage (Janeway and Medzhitov, 2002; Yi, 2016). This process is initiated when pattern recognition receptors (PRRs) recognize pathogen-associated molecular patterns (PAMPs) or damage-associated molecular patterns (DAMPs), leading to the production of pro-inflammatory cytokines such as interleukin (IL)-1 and tumor necrosis factor (TNF) (Medzhitov, 2008; Voet et al., 2019). While tightly regulated inflammatory responses are essential for maintaining immune homeostasis, dysregulated or chronic inflammation can lead to tissue damage and contribute to the development of various diseases, including cancer and autoimmune disorders (Scheiblich et al., 2020; Kaur et al., 2013; Park and Igarashi, 2013; Ham and Moon, 2013).

Among PRR-associated signaling pathways, inflammasomes are multiprotein complexes that amplify inflammatory responses through the activation of IL-1β (Zheng et al., 2020). The NLRP3 inflammasome is one of the most extensively studied inflammasomes and plays a critical role in a wide range of inflammatory diseases (Swanson et al., 2019; Ramachandran et al., 2024). NLRP3 activation occurs through a two-step process involving priming and activation (Lu and Wu, 2015; Kelley et al., 2019). The priming step is triggered by PAMPs, such as lipopolysaccharide (LPS), which activate nuclear factor kappa B (NF-κB) signaling and induce the expression of NLRP3 and pro-IL-1β (Ghonime et al., 2014). The activation step involves responses to DAMPs, including the generation of intracellular reactive oxygen species (ROS) (Martinon, 2010), endoplasmic reticulum stress, and K+ efflux (Zhou et al., 2020), leading to the assembly of the inflammasome complex and the release of active IL-1β (He et al., 2015). Given its central role in inflammatory signaling, the NLRP3 inflammasome has emerged as a key therapeutic target.

Despite extensive efforts, no Food and Drug Administration (FDA)-approved NLRP3 inhibitors are currently available, highlighting the need for alternative therapeutic strategies with improved efficacy and safety profiles. In this context, herbal medicines have gained increasing attention, as they contain diverse bioactive compounds capable of modulating multiple inflammatory pathways simultaneously (De Matos et al., 2024; Lee et al., 2024a; Lee et al., 2024b; Owida et al., 2025). Soyeom-Jetong (SJ) is a traditional polyherbal formulation composed of *Paeonia lactiflora Pallas*, *Glycyrrhiza inflata Batal*, and *Corydalis yanhusuo W.T. Wang*, which have been traditionally used for the management of inflammatory conditions and pain-related disorders. This combination is conceptually related to classical herbal prescriptions such as Jakyakgamcho-tang (Chinese name: Shaoyao Gancao Tang), a classical combination of *P. lactiflora* Pallas and *G. inflata* Batal, first described in the *Treatise on Cold Damage* (Eastern Han Dynasty, A. D 196∼219), under the Taiyang disease section, as well as Quzhang Tang described in Zhu’s Experiential Prescriptions (A.D. 1,266), which have been used for pain relief and musculoskeletal disorders.

Previous studies have demonstrated that key constituents of these herbs exhibit anti-inflammatory activities. For example, paeoniflorin, a principal component of *P. lactiflora* Pallas, has demonstrated anti-inflammatory activity by suppressing proinflammatory cytokine production and attenuating systemic inflammatory responses (Jiang et al., 2009; Liu et al., 2016). Glycyrrhizic acid, a major active compound of *G. inflata Batal,* has been reported to reduce inflammatory damage by suppressing ICAM-1, IFN-γ, iNOS, and COX-2 expression and by modulating MAPK signaling pathways (Li et al., 2014; Jurel et al., 2024; Wang and Du, 2016; Wang et al., 2023). In addition, corydalis-derived alkaloids have been shown to decrease the release of inflammatory cytokines such as TNF-α and IL-1β and to regulate neurotransmitter-related pathways associated with pain and inflammation (Tian et al., 2020; Alhassen et al., 2021; Zhang et al., 2025). Collectively, these findings suggest that the combined use of these herbs may exert synergistic anti-inflammatory effects through the modulation of multiple signaling pathways, including NF-κB- and MAPK-dependent inflammatory responses, as well as cytokine production and inflammasome-associated signaling. In addition, recent network pharmacology analyses have suggested that combinations of *P. lactiflora*, *G. inflata*, and *Corydalis yanhusuo* may regulate key inflammatory pathways, including NF-κB signaling and related immune responses (Kim YS. et al., 2024). However, despite these indications, experimental validation of their combined effects, particularly in relation to inflammasome-associated mechanisms, remains limited.

In this study, we aimed to investigate the anti-inflammatory effects of SJ using both *in vivo* and *in vitro* models and to determine whether SJ modulates NLRP3 inflammasome-related pathways. Using zebrafish and cellular assays, we sought to provide experimental evidence supporting the pharmacological activity of SJ and to explore its potential mechanism of action.

## Materials and Methods

### Preparation of SJ pharmacoacupuncture solution

The SJ used in the experiment was purchased from KSCP Co., Ltd. (Pocheon, Korea). Briefly, it is a pharmacoacupuncture solution manufactured by extracting and purifying the herbal medicine through freeze-drying. *Paeonia lactiflora* Pallas, *G. inflata* Batal, and *Corydalis yanhusuo* W.T. Wang that were manufactured to meet the quality standards of herbal medicines in Korean Pharmacopoeia and Korean Herbal Pharmacopoeia were purchased from Nature Sesang Co. (Pocheon, Republic of Korea). Dried *P. lactiflora* Pallas, *G. inflata* Batal, and *Corydalis yanhusuo* W.T. Wang were prepared in a 1:1:1 ratio of 450 g each. Approximately 6 L of 70% ethanol was added and extracted by double boiling using a heating mantle (MS-C408, Misung Scientific Co., Yangju, Republic of Korea) at 85 °C for 2 h 30 min. After cooling, the extract solution was filtered through a filter paper (grade; No 3, pore size; 3.0 μm, material; alpha-cellulose, thickness; 0.83mm; Hyundai Micro Co., Ltd., Seoul Korea) and concentrated (50 °C, 400 mbar, 60 rpm) using a rotary evaporator (Hei-VAP Expert Control ML G3 XL, Heidolph, Germany). Next, 80% and 90% ethanol treatment was performed, and after cooling, filtering was performed with a filter paper (1 μm, 150 mm), followed by secondary concentration (50 °C, 400 mbar, 60 rpm). After concentration, it was treated with 80% or 90% fermented alcohol (Prethanol A, Deoksan General Science Co., Seoul, Republic of Korea), cooled, filtered with filter paper (1 μm, 150 mm), and concentrated a second time (50 °C, 400 mbar, 60 rpm). Next, the solution was refiltered with filter paper (0.6–1.5 μm, 150 mm), and bacteria were removed using a filter integrity tester (Sartocheck 5PLus, Sartorius Korea Biotech Co., Seongnam, Republic of Korea) (filter specification, 0.1 μm × 4 inch capsule; diffusion test, maintain 4 bar; required value, 4.0 mL/min or less). The filtrate was freeze-dried in a freeze dryer (FDUT-8612, Operon Co., Gimpo, Korea) to obtain the final extract (89.2 g, 6.6% yield). The yield was calculated on a dry weight basis using the following formula: yield (%) = [weight of final freeze-dried extract (g)/total weight of raw herb material (g)] × 100. The initial amount of raw herbal material was 1,350 g, and the final freeze-dried powder obtained was 89.2 g. The process for manufacturing the pharmacoacupuncture solution is as follows: Add the freeze-dried powder (133.4 g) and sterile water for injection (22.5 L) to the stirring tank, stir for 30 mins at 20–25 °C, and check the pH condition (4.5–6.5). Maintain the stirring tank pressure at 1–1.5 bar and perform bacteria removal twice (filter specification, 0.2 μm × 5 inch capsule; diffusion test, maintain 2.8 bar; required value, 13 mL/min or less). The completed solution is filled into a vial and the cap is sealed. After post-sterilization (conditions: 122 °C, 25 min) using a high-pressure steam sterilizer, the product is packaged after conducting tests on appearance, capacity, specific gravity, pH, endotoxin, airtightness, and sterility.

### Quantification of standard compounds in freeze-dried SJ

To analyze the quantity of standard compounds in SJ, the sample was freeze-dried. The standards of glycyrrhizic acid, albiflorin, and paeoniflorin were provided by the Ministry of Food and Drug Safety (MFDS, Osong, Korea). The freeze-dried powder and the three standards were dissolved in distilled water (DW) and used for quantitative analysis. Ultra-performance liquid chromatography (UPLC) was performed using the ACQUITY UPLC system (Waters, MA, United States) equipped with an ACQUITY PDA detector. The sample and standards were analyzed under the following conditions: ACQUITY UPLC BEH Shield RP18 column (2.1 × 100 mm, 1.7 μm); column temperature, 40 °C; flow rate, 0.3 mL/min; and injection volume, 3 μL. For glycyrrhizic acid analysis; the mobile phase consisted of DW, acetonitrile and acetic acid (56:40:4, v/v/v) in isocratic mode, and chromatograms were monitored at 254 nm. For albiflorin and paeoniflorin analysis, the mobile phase consisted of 0.1% formic acid in DW (solvent A) and 0.1% formic acid in acetonitrile (solvent B) in a gradient mode (10% B, 0-5 min; 10%–20% B, 5-10 min), and chromatograms were monitored at 230 nm. Compound identification was performed by comparing retention times and UV spectra with those of authentic reference standards.

### Zebrafish maintenance

All animal experiments were conducted following the Institutional Animal Care and Use Committees (IACUC: KHNMC AP 2024-009) at Kyung Hee University Hospital in Gangdong. Adult TAB5 wild-type ABs were isolated overnight in fresh fish water at the appropriate pH (6.8 - 7.5) and were released from the divider the next morning to yield an embryo after mating. The embryos were incubated in MB-E3 solution until 1 day post-fertilization (dpf). At 1 dpf, embryos were dechorionated using pronase (Roche) and were incubated in 1-phenyl 2-thiourea (PTU, Sigma) until one or three dpf, depending on the experiment. All zebrafish embryos in this study were cultivated in the incubator at 28.5 °C to complete the experiments in the whole process.

### Zebrafish survival curve and drug treatment of immersion

To stabilize one dpf embryo after dechorionation, one dpf embryo was incubated in PTU for two hours before the next experiment. To plot the survival curve, the embryo was cultured in SJ containing 0, 0.05, 0.1, 0.5, 1, 5, and 10 mg/mL concentration of SJ from 1dpf up to five dpf, respectively, and each group of 30 embryos was cultured in 6 mL of solution in a 60 mm Petri dish. These treatment solutions are replaced every 24 h and the number of deaths and the number of emergences of edema phenotype are recorded. The phenotype pictures of the 3dpf embryo were illuminated under a 4x microscope (Leica M205FCA). Immersion experiments in which embryos were treated in different concentrations of SJ with or without 10 μg/mL LPS (Sigma, United States) were performed as in previous experiments. Every group was soaked in a 60 mm Petri dish containing 6 mL of treatment liquid, and the treatment solution was changed every 24 h. After 48 h of treatment, the samples, one dpf or three dpf embryo, were harvested in 4% Paraformaldehyde (PFA) solution at 4 °C ambient overnight for conservation.

### Sudan black B staining

45 mg of Sudan Black B powder (Sigma, United States) was dissolved in 25 mL of 70% ethanol and prepared as a stock solution after filtration. The working solution of Sudan Black B used for staining consisted of 10 mL stock solution, 40 mL 70% ethanol, and 50 μL Phenol mixed thoroughly and stored at room temperature. The depigmentation solution, consisting of 1% KOH, 1% H_2_O_2_, and DW, was made fresh for each experiment. Three dpf embryos held overnight in 4% PFA refrigerated storage for immersion or caudal amputation were removed and samples were washed three times at 4 °C with phosphate-buffered saline (PBS) after rocking to remove the liquid. After adding Sudan Black B working solution and rocking for 1 h away from light, these were washed three times with 70% ethanol. Then washed successively with PBS-Tween, the depigmentation solution, and PBS-Tween and finally stored in PBS solution at 4 °C until ready for imaging. Images were photographed by a microscope. The results of the experiments in terms of the area in the caudal hematopoietic tissue (CHT) section or caudal injury section and the number of neutrophils were quantified by ImageJ. Statistical analyses and graphing were performed by GraphPad Prism 9.

### Whole-mount *in situ* hybridization

After dehydration and rehydration, three dpf embryos were permeabilized with 99.7% acetone (Samchun, Korea) at −20 °C for 30 min. Then samples were kept in a hybridization buffer (containing 50% formamide, 5 × SSC, Torula yeast tRNA, heparin, 0.1% Tween-20, and 9 mM citric acid (pH 6.5)) in a 65 °C water bath for 2 h. Hybridize with Digoxigenin (DIG)-labeled RNA probes and incubate overnight. Embryos were washed in Hybridization Buffer/SSC mixture and SSC/PBST mixture and incubated with alkaline phosphate-conjugated DIG antibody (1:5,000, Roche, Basel, Switzerland) incubated overnight at 4 °C. The alkaline phosphatase (AP) buffer was manufactured from 100 mM Tris, pH 9.5, 50 mM MgCl2, 100 mM NaCl and 0.1% Tween-20. After several PBST washing procedures, antibody staining was shown after incubation in AP buffer and BCIP/NBT substrate (Promega, United States), and staining was completed using PBS/EDTA (pH 5.2) to stop the development.

### Caudal amputation

After incubation to three dpf in PTU, embryos were anesthetized with 1x Tricaine (Sigma, United States), and the caudal region was partially amputated under a microscope. Amputation embryos after treatment, with SJ solutions containing different concentrations for six hours, were fixed in 4% PFA and kept overnight. Images were visualized by a microscope (Leica M205FCA). Experimental pictures of neutrophils at the wound were obtained by quantifying the highly dense area of neutrophils and the number of neutrophils through ImageJ. Statistical analysis and graphing were performed using GraphPad Prism 9.

### cDNA synthesis and quantitative PCR

After 48 h of SJ treatment, 20 embryos were collected per biological replicate and stored at −80 °C until RNA extraction. Three independent biological replicates were performed for qPCR analysis. To prevent RNA degradation, RNA extraction was performed on ice using Trizol reagent (Sigma-Aldrich, United States), followed by DNase treatment (Biolabs) to remove residual DNA. Finally, cDNA was synthesized from 4 μg of total RNA by SuperScript IV reverse transcriptase (Invitrogen), and RNA concentrations were measured with a NanoDrop One spectrophotometer (Thermo Fisher Scientific). qPCR was performed using the Applied Biosystems StepOnePlus™ Real-Time PCR System (Thermo Fisher Scientific) with powerSYBR green PCR Master mix (Applied Biosystems). The expression levels of IL-1β, IL-6, and NLRP3 were quantified using the comparative threshold (Δ/Δ Ct) method and normalized to β-actin, which served as the endogenous control. Specific primer sequences were as follows: for β-actin, forward: 5′-CGA​GCA​GGA​GAT​GGG​AAC​C-3′ and reverse: 5′-CAA​CGG​AAA​CGC​TCA​TTG​C-3’; for IL-1β, forward 5′-GTC​ACA​CTG​AGA​GCC​GGA​AG-3′ and reverse 5′-GCA​GGC​CAG​GTA​CAG​GTT​AC-3’; for IL-6, forward: 5′-GCT​ATT​CCT​GTC​TGC​TAC​ACT​GG-3′ and reverse 5′-TGA​GGA​GAG​GAG​TGC​TGA​TCC-3’; for NLRP3, forward 5′-TGA​ACA​GGT​TGA​TGA​CTG​ATA​TGC​T-3′ and reverse: 5′-ACA​GCG​ATT​TTC​CCA​GCA​TCC​TTG​C-3’. Expression levels were calculated, and ANOVA statistics were analyzed and plotted by GraphPad Prism 9.

### Cell culture reagent and antibodies

Penicillin-streptomycin, fetal bovine serum (FBS), Roswell Park Memorial Institute (RPMI) 1,640 and Dulbecco’s modified Eagle medium (DMEM) were purchased from GenDEPOT. Antibodies against alpha-tubulin (sc-5286) was procured from Santa Cruz Biotechnology. Human IL-1β (AF-401-NA) and mouse IL-1β antibodies (AF-201-NA) were purchased from R&D Systems. LPS from *Porphyromonas gingivalis* (Ultrapure) (tlrl-ppglps), nigericin (tlrl-nig), ATP (tlrl-atpl) and flagellin (FLA-ST; tlrl-epstfla) were procured from InvivoGen. Lipofectamine 3000 (L3000075) and MitoProbe™ JC-1 Assay Kit (M34152) were purchased from Invitrogen. Lipidofect-P (LDL-P001) was procured from lipidomia. Phorbol 12-myristate 13-acetate (PMA; HY-18739) was obtained from MedChem Express. EZ-Cytox (EZ-500) was purchased from DoGenBio. N-acetyl-l-cysteine (NAC; A9165-25G) was purchased from Sigma-Aldrich. Xpert Protease Inhibitor Cocktail Solution (#P3100-020) was obtained from GenDEPOT. The ROS assay kit (DCFDA/H2DCFDA) (ab113851) and nuclei stained with mounting medium with DAPI (ab104139) were purchased from Abcam. Bright-Glo™ luciferase assay system (E2610) and pGL4.32 luciferase reporter vector (E8491) were procured from Promega.

### Cell culture and stimulation

J774 A.1 and THP-1 cell line were cultured by datasheet on ATCC website (Anonymous author, 2023a; Anonymous author, 2023b). To differentiate THP-1 cell line, THP-1 was treated with PMA (500 nM) for 3 h, and then incubated for 2 days. Both are primed by LPS (100 ng/mL) for 3 h and then treated with SJ (1, 2.5, 5, and 10 mg/mL) for 2 h. Then, to activate NLRP3 inflammasome, THP-1 was treated with nigericin (10 μM) for 1 h and J774 A.1 was treated with ATP (5 mM) for 30 min respectively. To activate AIM2 or NLRC4 inflammasome, J774 A.1 was transfected with dsDNA (1 μg) or flagellin (50 μg/mL) and then incubated for 3 h. All *in vitro* experiments were performed in triplicate (n = 3).

### Cell viability assay

Differentiated THP-1 cells (0.5 × 10^5^ cells/well) were incubated in a 96-well plate for 2 days before the experiment. J774 A.1 cells (0.5 × 10^5^ cells/well) were incubated in a 96-well plate for 1 day before the experiment. These cells were treated with SJ (1, 2.5, 5 and 10, and 20 mg/mL) for 2 h. And then, these cells were treated with EZ-cytox for 1 h. Using the iMark™ Microplate Absorbance Reader (Bio-Rad, 1681130), Cell viability was measured at 450 nm wavelength. Data are presented as the mean ± SEM. Statistical analyses were performed using one-way ANOVA with Bonferroni *post hoc* testing (****P < 0.0001).

### NF-κb luciferase assay

293 F T cells (0.2 × 10^5^ cells/well) were seeded in a 96-well white plate and incubated overnight. Then, pGL4.32 luciferase reporter vector (0.1 μg/well) was transfected to 293 F T cells using lipidofect-P and then transfected 293 F T cells were incubated overnight. Transfected 293 F T cells were treated by TNF-α (20 ng/mL) for 5 h with or without SJ (1, 5, and 10 mg/mL). The Luminescence was measured using a BMG LabTech FLUOstar OPTIMA microplate reader. Data are presented as mean ± SEM. Statistical analyses were performed using one-way ANOVA with Bonferroni *post hoc* testing (ns = not significant).

### Mitochondrial membrane potential measurement

J774 A.1 cells (2 × 10^5^ cells/well) were seeded in a four well culture plate and incubated overnight. The cells were primed with LPS (100 ng/mL) and subsequently treated with or without SJ (5 and 10 mg/mL) for 2 h. After treatment, the cells were stained with JC-1 dye (10 μM), at 37 °C in the dark for 10 min. The cells were then activated with ATP (5 mM) for 5 min. Mitochondrial membrane potential was measured using a confocal laser-scanning microscope (Carl Zeiss, LSM710).

### ASC oligomerization assay

THP-1 cells were lysed using lysis buffer, and the resulting pellets were resuspended in PBS. The pellets were then treated with disuccinimidyl suberate (DSS) (2.5 mM) for 30 min at 25 °C for cross-linking. After that, they were separated by SDS-PAGE and analyzed by immunoblotting.

## Results

### UPLC-based identification and quantification of marker compounds in SJ

**FIGURE 1:**
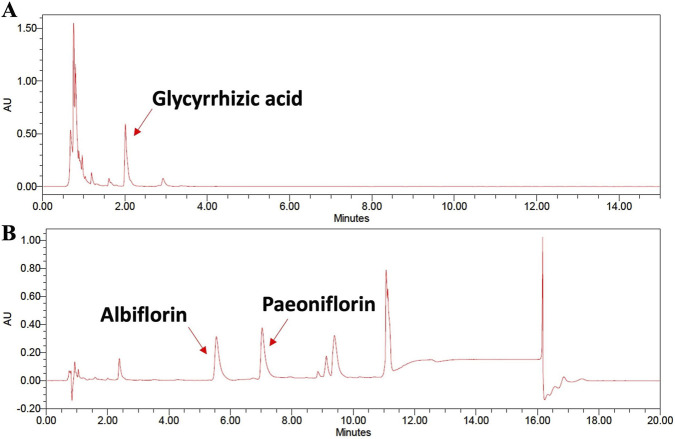
Quantitative analysis of Soyeom-Jetong (SJ) using ultra-performance liquid chromatography (UPLC). **(A)** UPLC chromatogram of freeze-dried SJ showing glycyrrhizic acid. **(B)** UPLC chromatogram showing albiflorin and paeoniflorin **(B)**. The peaks corresponding to each compound are highlighted and show the presence and quantity of each compound in the sample. The x-axis represents retention time (min), and the y-axis represents intensity (AU).

To assess the quality and batch consistency of SJ, three representative pharmacopoeial marker compounds—glycyrrhizic acid, albiflorin, and paeoniflorin—were analyzed by UPLC. These compounds were selected as representative quality-control markers, and the targeted UPLC analysis was optimized for their identification and quantification in SJ. Using authentic standards, the presence of these compounds was confirmed in the freeze-dried SJ formulation. The UPLC results are presented as two sub-figures, because the analytical conditions were optimized separately for glycyrrhizic acid and albiflorin/paeoniflorin (Figure 1). Target compounds were identified by matching their retention times and UV spectra to those of the standards. Quantitative analysis using standard calibration curves showed that the concentrations of glycyrrhizic acid, albiflorin, and paeoniflorin were 29.18, 53.01, and 66.05 mg/g, respectively. These findings confirm that the three representative pharmacopoeial marker compounds were successfully identified and quantified in the SJ formulation.

### Dose-dependent effects of SJ on zebrafish survival and developmental phenotypes

We used a zebrafish (*Danio rerio*) animal model to investigate the effects of SJ. Zebrafish have become indispensable for inflammation research because of their unique characteristics (Renshaw et al., 2006; Xie et al., 2020; Smith, 2024). Transparent embryos and their rapid development are ideal for studying inflammatory response dynamics. Zebrafish possess both innate and adaptive immune systems that effectively simulate human inflammatory processes (Belo et al., 2021; Leiba et al., 2023). The development of transgenic lines has facilitated real-time observation of inflammatory cells such as neutrophils and macrophages (Balde et al., 2024). In addition, zebrafish express various inflammatory genes, including TNF-α, TNF-β, IL-6, and IL-1β, with expression patterns that closely mimic those in mammals, thus enhancing their value in investigating gene regulation and signaling pathways in inflammation (Denans et al., 2022; Ollewagen et al., 2024).

**FIGURE 2:**
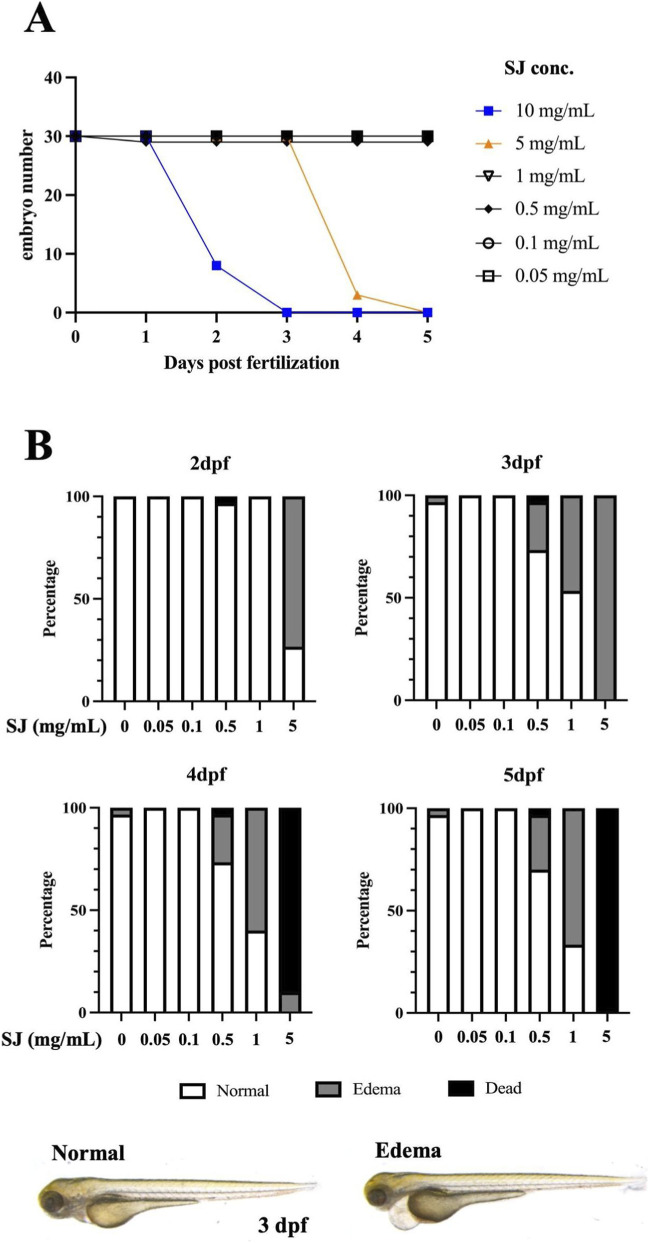
Toxicity assessment of SJ in zebrafish using survival rates and phenotypic observations. **(A)** Survival rate of zebrafish larvae treated with SJ from one dpf to five dpf (SJ concentrations: 0, 0.05, 0.1, 0.5, 1, 5, and 10 mg/mL; n = 30 for each concentration). Larvae up to five dpf could survive at concentrations of 1 mg/mL SJ or below. **(B)** Phenotypic observations of cardiac edema in zebrafish larvae treated with SJ. Cardiac edema was evaluated in 30 larvae from two to five dpf across 0-5 mg/mL SJ. The white, gray, and black bars represent normal, cardiac edema, and death, respectively. Representative images of cardiac edema and normal conditions are also shown.

To evaluate the tolerance of SJ, zebrafish larvae at 1 day post-fertilization (dpf) were incubated with SJ at concentrations of 0, 0.05, 0.1, 0.5, 1, 5, and 10 mg/mL until five dpf (Figure 2A). At 10 mg/mL, all larvae died by three dpf, whereas exposure to 5 mg/mL resulted in significant mortality by four dpf and complete mortality by five dpf. In contrast, larvae treated with lower concentrations survived throughout the observation period. Larvae exposed to concentrations above 0.5 mg/mL exhibited an edematous phenotype (Figure 2B). The incidence of edema was evaluated every 24 h following SJ treatment, revealing a dose-dependent increase. These findings indicate that concentrations above 0.5 mg/mL may induce toxic effects. Conversely, no significant morphological or physiological abnormalities were observed at concentrations up to 0.1 mg/mL, suggesting that this range is suitable for evaluating the pharmacological effects of SJ without confounding toxicity.

### SJ reduces neutrophil accumulation in LPS-induced zebrafish inflammation models

**FIGURE 3:**
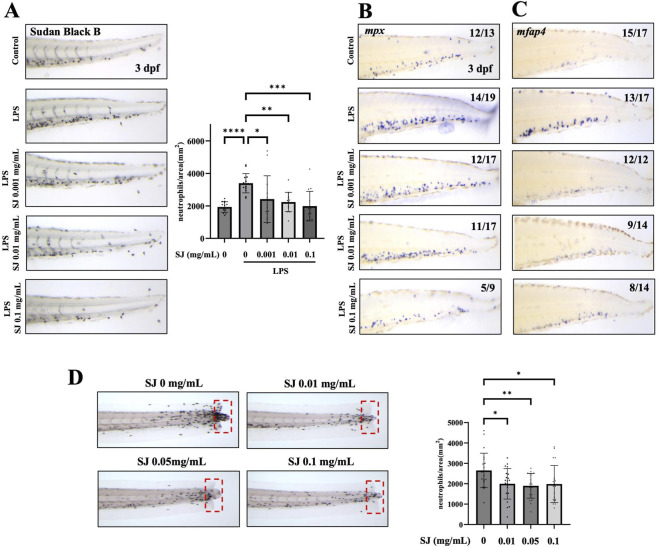
SJ exerts anti-inflammatory effects in zebrafish embryos with LPS-induced inflammation. **(A)** Changes in neutrophil counts in the CHT region stained with Sudan Black B following LPS induction. Larvae were exposed to 0 (control), 0.001, 0.01, and 0.1 mg/mL SJ. Bar graph showing the mean number of neutrophils in the CHT region (0 mg/mL, n = 14; 0 mg/mL + LPS, n = 14; 0.001 mg/mL + LPS, n = 14; 0.01 mg/mL + LPS, n = 15; 0.1 mg/mL + LPS, n = 14). **(B,C)** Representative images of WISH results for neutrophil (*mpx*) and macrophage (*mfap4*) markers in three dpf embryos treated with 0.001, 0.01, and 0.1 mg/mL SJ. Numbers at the top right of each image indicate the number of embryos showing the representative phenotypes relative to the total number of embryos examined. **(D)** Neutrophil counts near injury sites in embryos at 3 days post-amputation (dpa) following treatment with 0.01, 0.05, and 0.1 mg/mL SJ. Bar graph showing the mean number of neutrophils within a 0.03 mm^2^ area (red-dotted box) near the injury site (0 mg/mL, n = 22; 0.01 mg/mL, n = 21; 0.05 mg/mL, n = 21; 0.1 mg/mL, n = 20). Data are presented as mean ± SEM. Statistical analyses were performed using one-way ANOVA (*P < 0.05, **P < 0.01, ***P < 0.001, ****P < 0.0001).

To investigate the role of SJ in modulating inflammatory response, an inflammation model was established by treating zebrafish larvae with 10 μg/mL LPS. To assess the inflammatory effects, Sudan Black B staining was performed to quantify the average number of neutrophils in the caudal hematopoietic tissue (CHT), where embryonic blood cells form and mature (Lee et al., 2024b). Interestingly, in three dpf embryos co-administered LPS and SJ, the distribution and number of neutrophils in the CHT were reduced, suggesting anti-inflammatory activity of SJ (Figure 3A). In these LPS-exposure experiments, low concentrations of SJ (0.01 and 0.1 mg/mL), which did not elicit developmental or survival toxicity, significantly reduced the average neutrophil counts in the CHT under inflammatory conditions. Furthermore, whole-mount *in situ* hybridization (WISH) revealed downregulation of the neutrophil marker *mpx* (Figure 3B). In addition, expression of the macrophage marker *mfap4* was also decreased, indicating that SJ reduced the LPS-induced activation of both neutrophils and macrophages (Figure 3C). To further investigate the direct effects of SJ on neutrophil dynamics, physically wounded three dpf embryos underwent caudal amputation (Figure 3D). At 6 h post-amputation, all SJ-treated groups exhibited reduced neutrophil counts at the wound site compared to the untreated group, indicating the direct inhibitory effects of SJ on inflammation at the injury site.

**FIGURE 4:**
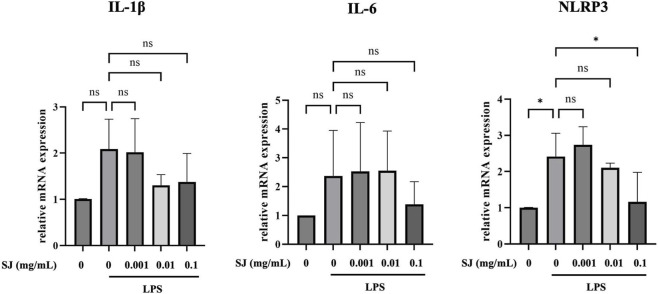
Examination of inflammation markers in zebrafish embryos treated with SJ. qPCR analysis of the expression levels of inflammatory marker genes, including IL-1β, IL-6, and the inflammasome-related gene NLRP3, in three dpf zebrafish embryos. Embryos were treated with LPS in the presence or absence of SJ for 48 h prior to analysis. Gene expression levels were normalized to the control group. Data are presented as mean ± SEM from three independent biological replicates (n = 3), each consisting of 20 embryos. Statistical significance was determined using one-way ANOVA (ns, not significant; *P < 0.05).

To further understand the mechanism underlying the anti-inflammatory effects of SJ, quantitative real-time PCR (qPCR) was employed to measure the expression levels of inflammatory markers, including IL-1β, IL-6, and NLRP3, in zebrafish larvae. At 0.1 mg/mL SJ, where the increased neutrophil count was suppressed, a decreasing trend in IL-1β and IL-6 expression was observed compared with LPS-induced control groups; however, these changes were not statistically significant (Figure 4). Additionally, NLRP3 expression was investigated, as the NLRP3 inflammasome plays a key role in the activation and release of proinflammatory cytokines and is among the most extensively studied inflammatory pathways (Blevins et al., 2022). Interestingly, LPS-induced NLRP3 expression was reduced following SJ treatment. These findings suggest that SJ may modulate inflammation-related pathways, including NLRP3-associated signaling.

### SJ suppresses NLRP3 inflammasome activation *in vitro*

**FIGURE 5:**
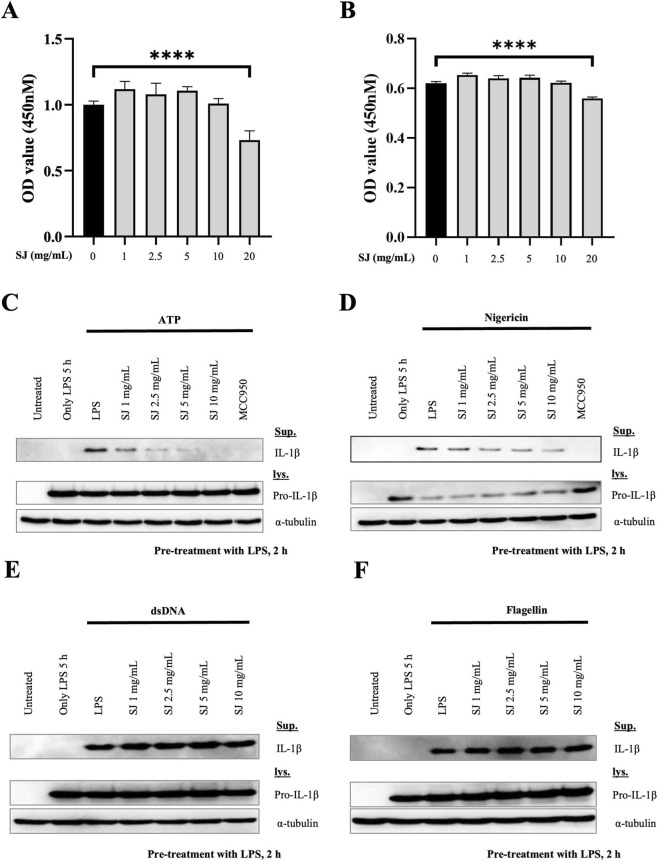
SJ inhibits the NLRP3 inflammasome activation *in vitro*. **(A,B)** Assessment of cell survival following SJ treatment *in vitro*. **(A)** J774 A.1 cells were treated with SJ (1, 2.5, 5, 10, and 20 mg/mL) for 2 h and subsequently treated with EZ-cytox for 1 h. **(B)** THP-1 cells were differentiated with PMA (500 nM) for 3 h. PMA-differentiated THP-1 cells were treated with SJ (1, 2.5, 5, 10, and 20 mg/mL) for 2 h and subsequently treated with EZ-cytox for 1 h. **(C)** J774 A.1 cells were primed with LPS (100 ng/mL) for 3 h and then treated with SJ (1, 2.5, 5, and 10 mg/mL) for 2 h. LPS-primed J774 A.1 cells were activated with ATP (5 mM) for 30 min, with or without MCC950 (100 nM). **(D)** PMA-differentiated THP-1 cells were primed with LPS (100 ng/mL) for 3 h and then treated with SJ (1, 2.5, 5, and 10 mg/mL) for 2 h. LPS-primed THP-1 cells were activated using nigericin (10 μM) for 1 h, with or without MCC950 (100 nM). **(E,F)** J774 A.1 cells were primed with LPS (100 ng/mL) for 3 h and then treated with SJ (1, 2.5, 5, and 10 mg/mL) for 2 h. LPS-primed J774 A.1 cells were transfected with dsDNA (2 μg/mL) or flagellin (1.25 μg/mL) for 3 h. Supernatants and lysates were analyzed using immunoblotting. Data are presented as mean ± SEM from three independent experiments (n = 3). Statistical analyses were performed using one-way ANOVA with Bonferroni *post hoc* testing (****P < 0.0001).

To further investigate the anti-inflammatory effects of SJ observed in the zebrafish model, we performed *in vitro* experiments to assess whether SJ inhibits NLRP3 inflammasome-mediated IL-1β secretion. Initially, the effect of SJ treatment on cell viability was evaluated. SJ exhibited minimal cytotoxicity in both J774 A.1 and THP-1 cells, maintaining cell viability above 90% at concentrations up to 10 mg/mL. Significant cytotoxicity was observed at 20 mg/mL; therefore, 10 mg/mL was selected as the maximum concentration for subsequent experiments (Figures 5A,B). SJ was evaluated at concentrations of 1, 2.5, 5, and 10 mg/mL to assess its dose-dependent effects across a broad range. As the active inflammatory cytokine IL-1β is secreted when the NLRP3 inflammasome is activated, we evaluated whether SJ could suppress IL-1β secretion, as a measure of its anti-inflammatory potential. Western blotting was used to quantify IL-1β levels in two cell lines, J774 A.1 and THP-1, following treatment with SJ. SJ treatment significantly suppressed the secretion of IL-1β in a dose-dependent manner (Figures 5C,D). At the highest concentration, SJ treatment resulted in an IL-1β reduction similar to that of MCC950, a specific inhibitor of the NLRP3 inflammasome (Bakhshi and Shamsi, 2022; Li et al., 2022). Interestingly, while SJ effectively suppressed IL-1β secretion, the expression of pro-IL-1β and α-tubulin was unaffected, suggesting that SJ selectively targets inflammasome activation without influencing upstream processes.

To determine whether the reduction in IL-1β production by SJ is specifically mediated through the NLRP3 inflammasome rather than a general effect on inflammasome pathways, we evaluated its effects on AIM2 and NLRC4 inflammasomes. In J774 A.1 cells, the AIM2 and NLRC4 inflammasomes were activated by transfection with double-stranded DNA (dsDNA) and flagellin, respectively (Sharma et al., 2019; Zhao et al., 2011). IL-1β secretion was measured following SJ treatment to evaluate its effect on AIM2 and NLRC4 inflammasome-mediated inflammatory responses. SJ treatment did not reduce the IL-1β secretion mediated by the AIM2 and NLRC4 inflammasomes (Figures 5E,F). These findings indicate that SJ suppresses IL-1β secretion without affecting the upstream priming step, as evidenced by unchanged pro-IL-1β expression. In addition, the lack of effect on AIM2 and NLRC4 inflammasomes supports a selective inhibitory effect of SJ on the NLRP3 inflammasome. Collectively, these results suggest that SJ preferentially suppresses NLRP3 inflammasome activation under the tested conditions.

### SJ suppresses ASC oligomerization without affecting NF-κB activation or mitochondrial ROS levels

**FIGURE 6:**
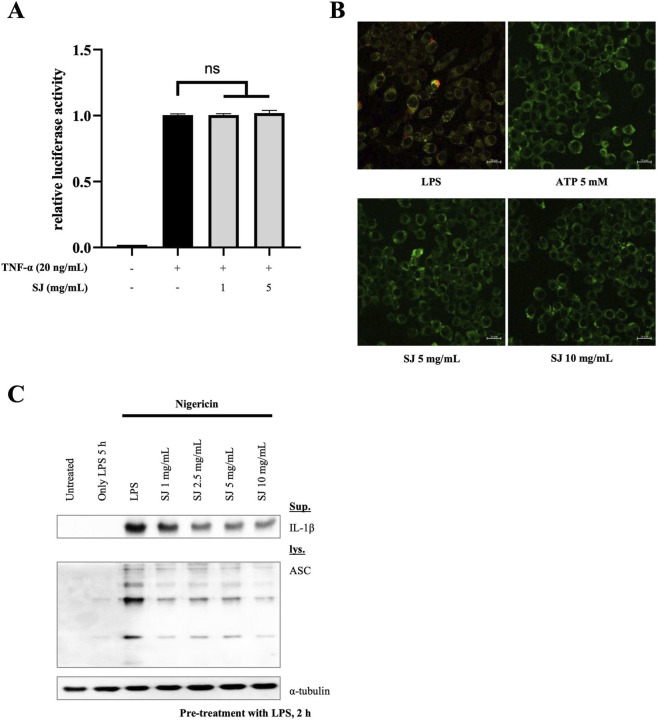
SJ suppresses NLRP3 inflammasome activation by suppressing ASC oligomerization. **(A)** 293FT cells were transfected with pGL4.32 luciferase reporter vector (0.1 μg/well). Transfected 293FT cells were activated with TNF-α (20 ng/mL) and treated with SJ (1, 5, and 10 mg/mL) simultaneously for 5 h. The luciferase activity of NF-κB was measured using a Promega Bright-Glo luciferase assay system. **(B)** LPS-primed J774A.1 cells were treated with SJ (5 and 10 mg/mL) and stained with JC-1 dye (10 μM) for 10 min. ATP (5 mM) was used to activate mitochondrial membrane depolarization for 5 min. The mitochondrial membrane potential was analyzed using the MitoProbe™ JC-1 Assay Kit. **(C)** PMA-differentiated THP-1 cells were primed with LPS (100 ng/mL) for 3 h and then treated with SJ (1, 2.5, 5, and 10 mg/mL) for 2 h. LPS-primed THP-1 cells were activated using nigericin (10 μM) for 1 h. ASC oligomerization was analyzed by DSS cross-linking, followed by immunoblotting. Data are presented as mean ± SEM from three independent experiments (n = 3). Statistical analyses were performed using one-way ANOVA with Bonferroni *post hoc* testing (ns, not significant).

To determine whether SJ is involved in the priming step of the NLRP3 inflammasome, we performed luciferase assays to evaluate its effect on NF-κB signaling. TNF-α stimulation significantly increased luciferase activity, confirming activation of the NF-κB pathway. However, treatment with SJ (1 and 5 mg/mL) did not significantly suppress NF-κB activity (Figure 6A). As shown in Figures 5C,D, SJ reduced IL-1β production following NLRP3 activation, suggesting that SJ does not affect the priming step but instead acts at the activation stage of the NLRP3 inflammasome.

As mitochondrial ROS are critical in the activation of the NLRP3 inflammasome (Tschopp and Schroder, 2010), we investigated whether SJ influenced mitochondrial ROS production. To assess mitochondrial ROS levels, we measured the mitochondrial membrane potential (MMP), since alterations in MMP are closely associated with mitochondrial ROS generation. Excessive mitochondrial ROS production is typically accompanied by MMP depolarization; therefore, MMP assessment provides an indirect but reliable indicator of mitochondrial ROS levels. Compared with ATP treatment alone, SJ treatment did not significantly alter mitochondrial depolarization, as indicated by the red-to-green fluorescence intensity ratio (Figure 6B). These findings suggest that SJ did not significantly affect mitochondrial ROS levels.

To further define the step affected by SJ during NLRP3 inflammasome activation, we examined the oligomerization of the apoptosis-associated speck-like protein containing a caspase recruitment domain (ASC), a critical step in inflammasome assembly (Fu and Wu, 2023). Upon activation, NLRP3 recruits the adaptor protein ASC, leading to ASC oligomerization and the formation of a multiprotein complex required for caspase-1 activation and subsequent IL-1β maturation. SJ significantly inhibited ASC oligomer formation following NLRP3 activation (Figure 6C). These findings indicate that SJ acts at the inflammasome assembly stage by suppressing ASC oligomerization, thereby reducing NLRP3 inflammasome activation.

## Discussion

Our study explored the anti-inflammatory potential of an SJ extract mixture that included *P. lactiflora* Pallas, *G. inflata* Batal, and *Corydalis yanhusuo* W.T. Wang. SJ has been used as a pharmacopuncture formulation for pain- and inflammation-related musculoskeletal conditions, and network pharmacology analysis has suggested its potential association with inflammatory signaling pathways (Kim YS. et al., 2024; Chae et al., 2023; Kim JY. et al., 2024). However, experimental evidence supporting its anti-inflammatory mechanisms has remained limited. The component herbs of SJ are well known in traditional medicine for their therapeutic properties and may contribute collectively to its pharmacological activity. *Paeonia lactiflora* Pallas is rich in active ingredients, such as paeoniflorin, paeoniflorin glycoside, and paeonol, which inhibit NF-κB signaling and NLRP3 inflammasome activation while acting as antioxidants to maintain cell membrane integrity (Ma et al., 2018; Wankun et al., 2011). *Glycyrrhiza inflata* Batal contains glycyrrhizin, glycyrrhetinic acid, and flavonoids, which are known for their robust anti-inflammatory effects in various inflammatory diseases and act by suppressing the NF-κB signaling pathway, demonstrating antioxidant properties, and inhibiting COX-2 and iNOS expression (Asl and Hosseinzadeh, 2008; Kao et al., 2014; Haraguchi et al., 1998; Wang and Nixon, 2001). *Corydalis yanhusuo* W.T. Wang contains alkaloids, such as tetrahydropalmatine, protopine, and dehydrocorydaline, which have been associated with analgesic and anti-inflammatory effects and may act by inhibiting MAPK signaling and reducing the release of inflammatory factors such as TNF-α and IL-1β (Bao et al., 2021; Feng et al., 2023; Wu et al., 2021).

Despite its use in clinical acupuncture, the precise mechanism underlying the anti-inflammatory effects of SJ remains unclear. In this study, we used an inflammation-induced zebrafish model and *in vitro* assays to demonstrate that SJ reduces inflammation. Importantly, zebrafish larvae exposed to high SJ concentrations developed cardiac edema and exhibited increased mortality, indicating dose-dependent toxicity. These findings suggest that the safety profile of SJ is concentration-dependent, with higher concentrations associated with adverse effects such as lethality and edema. In contrast, anti-inflammatory effects were observed at concentrations that did not induce obvious developmental abnormalities or survival toxicity in zebrafish. At these concentrations, SJ significantly reduced neutrophil accumulation in LPS-induced inflammation models and caudal injury assays. This was further supported by molecular analysis showing reduced NLRP3-associated gene expression and decreasing trend in inflammatory cytokine expression following SJ treatment.

Although many inflammasomes directly detect DAMPs or PAMPs, the NLRP3 inflammasome is uniquely sensitive to changes in cellular homeostasis and does not require direct recognition of specific components. When cellular changes are detected, the NLRP3 inflammasome is activated, leading to IL-1β and IL-18 secretion. These cytokines amplify the inflammatory response by activating other proinflammatory cytokines, forming a positive feedback loop (Dinarello, 2009). NLRP3 is abnormally activated by various cellular changes, and this dysregulated activation exacerbates symptoms by causing excessive responses to cellular alterations associated with inflammatory diseases, thereby emphasizing its significance as a therapeutic target for managing these disorders.

Targeting the NLRP3 inflammasome has garnered significant attention in the development of therapeutic agents for inflammatory diseases, owing to its strong association with various pathological conditions (Yang et al., 2019). The direct inhibition of IL-1β has been a promising strategy but may increase susceptibility to infections, underscoring the need for more selective approaches (Tengesdal et al., 2024). Directly targeting NLRP3 offers the advantage of mitigating issues caused by its excessive activation while preserving IL-1β production necessary for infection defense, thereby addressing the vulnerability associated with IL-1β inhibition. Furthermore, the potential for the oral administration of NLRP3 inhibitors may improve practicality and patient compliance, supporting their continued development as therapeutic candidates for inflammatory disorders. MCC950, a specific NLRP3 inhibitor, effectively blocks the activation of the NLRP3 protein and has shown significant anti-inflammatory effects in various *in vivo* and *in vitro* models. Thus, it has been considered a potential treatment for a range of NLRP3-related diseases (Coll et al., 2015). However, its effectiveness does not extend to patients with CAPS harboring specific NLRP3 mutations (Vande Walle et al., 2019). In our study, SJ reduced NLRP3-associated inflammatory responses under the tested conditions. In particular, the additional ASC oligomerization analysis suggests that SJ affects the inflammasome assembly stage, providing mechanistic insight beyond the reduction of inflammatory cytokine production. However, the precise molecular target of SJ within the NLRP3 inflammasome pathway remains to be determined. Notably, although numerous small molecules targeting NLRP3 have shown promise for selectively suppressing NLRP3 inflammasome activation, none have received an FDA approval for clinical use. Although SJ has been used in traditional medicine, the present data do not establish its clinical safety or therapeutic efficacy. Therefore, SJ should be regarded as a candidate for further investigation rather than as an established therapeutic option for NLRP3-related inflammatory diseases.

The present findings also need to be interpreted in light of the chemical complexity of SJ. SJ is a polyherbal formulation containing multiple potentially bioactive constituents, and the anti-inflammatory effects observed in this study may reflect the combined activity of multiple components rather than a single compound. Consistent with this possibility, several representative constituents of the component herbs, including paeoniflorin, glycyrrhizic acid, and Corydalis-derived alkaloids, have been reported to possess anti-inflammatory activities (Jiang et al., 2009; Li et al., 2014; Tian et al., 2020). In the present study, glycyrrhizic acid, albiflorin, and paeoniflorin were quantified as representative pharmacopoeial marker compounds for quality assessment and batch consistency evaluation. However, this targeted analysis does not comprehensively reflect the full chemical composition of SJ or identify the active constituents responsible for its pharmacological effects. Therefore, further phytochemical profiling and bioactivity-guided studies are warranted to identify additional bioactive constituents associated with the anti-inflammatory effects of SJ.

Moreover, additional studies using mammalian *in vivo* models will be necessary to validate the efficacy, safety, and precise mechanisms of action of SJ. In particular, the dose-dependent toxicity observed in zebrafish highlights the need for careful dose optimization and further safety evaluation. Taken together, our findings provide experimental evidence that SJ reduces inflammatory responses in zebrafish and *in vitro* models and support its potential as an anti-inflammatory candidate for further investigation, while emphasizing the need for additional validation before therapeutic application.

## Conclusion

SJ, a traditional East Asian herbal formulation, showed anti-inflammatory activity by modulating neutrophil-mediated inflammation and suppressing NLRP3 inflammasome activation under the experimental conditions used in this study. Using both zebrafish and *in vitro* models, SJ reduced inflammatory responses under experimental conditions. In particular, SJ did not affect upstream NF-κB signaling or intracellular ROS levels, suggesting that its effects are associated with the inflammasome activation step. These findings provide experimental support for the pharmacological activity of SJ and suggest its potential as an anti-inflammatory candidate for further investigation.

## Data Availability

The original contributions presented in the study are included in the article; further inquiries can be directed to the corresponding author.
